# Rubber-related chemical contamination in milk: Implications for dairy production systems

**DOI:** 10.1016/j.fochx.2026.104373

**Published:** 2026-08-29

**Authors:** Florian Breider, Dominique Grandjean, Claire Andrey, Thibault Masset, Beat J. Brüschweiler

**Affiliations:** a Ecole Polytechnique Fédérale de Lausanne (EPFL), Central Environmental Laboratory, Station 2, CH-1015 Lausanne, Switzerland; b Federal Food Safety and Veterinary Office (FSVO), Knowledge Foundations Division, Bern, Switzerland

**Keywords:** Rubber, DPG, 6-PPD, 6-PPD-Q, Milk, Dairy products

## Abstract

Rubber-related chemicals are widely used in tires and technical rubber products, but their occurrence in food remains poorly understood. This study analyzed 17 raw milk samples and rubber components from dairy production systems for benzothiazoles, guanidine accelerators, and *p*-phenylenediamine antioxidants. Four compounds (S-BTH, DPG, 6-PPD, and 6-PPD-Q) were detected in milk (38.8–26,258.5 ng/L), with at least one quantified in 41% of samples (7/17), whereas none of the target compounds were detected in the three samples collected from alpine farms. Fourteen rubber-related chemicals were identified in milk-contact rubber components (42.9 μg/g–3.7 mg/g). Solubilization experiments confirmed partial leaching, with released chemical profiles reflecting bulk material composition. These findings indicate that rubber-related chemicals can occur in raw milk and that both environmental sources and migration from food-contact materials represent potential contamination pathways. However, the limited sample size and absence of a clear relationship with road traffic intensity preclude identification of the predominant contamination source.

## Highlights

• Rubber-related chemicals were detected in 41% of raw milk samples
• High concentrations of 14 rubber additives were identified in dairy rubber components
• Solubilization experiments demonstrated additive leaching from rubber materials

## 1 Introduction

Rubber-related compounds widely used in tires and various technical rubber products (e.g. soles, hoses, seals, etc.), represent a growing environmental concern due to their widespread dissemination and potential ecological and health implications. These compounds, which include a variety of additives such as vulcanizing agents, antioxidants, and plasticizers, are released into the environment as tires degrade.(Wang et al., 2024; Wang et al., 2025) Global emissions of tire and road wear particles (TRWPs) are estimated to reach approximately six million tons annually, introducing significant quantities of pollutants into agricultural systems through atmospheric deposition, road runoff, and biosolid fertilizers.(Baensch-Baltruschat et al., 2020; Evangeliou et al., 2020; Kole et al., 2017) Recent studies have underscored the environmental and health implications of tire-related compounds, particularly *N*-(1,3-dimethylbutyl)-*N*′-phenyl-1,4-benzenediamine (6-PPD) and its oxidation by-product 6-PPDQ.(Tian et al., 2020) Despite significant research into their environmental impact, their toxicity in mammals remains insufficiently understood. For instance, Fang et al. (2023) evaluated the effects of 6-PPD and 6-PPDQ on the livers of C57BL/6 mice through six weeks of oral administration. (Fang et al., 2023) Results showed dose-dependent accumulation in the liver, increased liver weight, and elevated triglyceride levels, suggesting hepatotoxicity. Gene expression analysis revealed disruptions in glycolipid metabolism, immune responses, and glutathione metabolism. Additionally, 6-PPDQ induced immune responses by activating immune-related genes and promoting macrophage infiltration. 1,3-diphenylguanidine (DPG) is a chemical registered under REACH and is produced or imported into the European Economic Area in quantities ranging from 10,000 to 100,000 tons annually. Bempong and Hall (1983) revealed that chronic exposure to DPG in hamsters and mice caused time- and dose-dependent reproductive abnormalities. (Bempong & Hall, 1983) These included altered sperm morphology, reduced sperm count, lower testicular weight, irregular seminiferous tubules, diminished spermatids and spermatozoa, decreased fertility indices, fewer implants per pregnancy, and increased fetal mortality. Recent studies by Bergmann et al. (2024) confirmed DPG's genotoxic potential using the *umuC* bioassay. (Bergmann et al., 2024) The potential toxicity of some rubber-related chemicals raises questions about possible exposure routes in animals and humans. The main potential routes of exposure are inhalation of fine TRWPs and ingestion of food contaminated with TWRPs or other technical rubber products such hoses or seals. Research is beginning to focus on the presence of these compounds in soils and edible plants. (Breider et al., 2025; Sartori et al., 2026) Recent research has demonstrated that rubber-related chemicals can be absorbed and metabolized by edible plants such as lettuce and carrots, raising concerns about their entry into the human food chain. (Castan et al., 2023; Sherman et al., 2024; Wu et al., 2024) Compounds like benzothiazoles (BTH), 6-PPD, and 6-PPDQ have been detected in various vegetables, with studies indicating that their lipophilicity plays a significant role in their uptake and translocation within plants. (Breider et al., 2025; Castan et al., 2023; Sartori et al., 2026) To our knowledge only Cao et al. (2026) reported the transfer of rubber-related chemicals into animal-based food products. (Cao et al., 2026) They identified the presence of *p*-phenylenediamines (PPDs) and *p*-phenylenediamine-derived quinones (PPDQs) in chicken eggs from 13 countries in Europe, Asia, Oceania and North America. (Cao et al., 2026) With concentrations ranging from 32.5 to 3.30 × 10^3^ ng/kg of egg yolk, 6-PPDQ was the most abundant PPDQ detected in eggs. The widespread detection, with notably high concentrations and detection frequencies in egg samples, underscores an important yet underappreciated pathway for human exposure to these rubber-derived contaminants.

Milk from animals grazing on crops grown on contaminated soil or inhaling fine TRWPs may represent an additional and largely overlooked route of human exposure to rubber-related compounds. Despite growing evidence of environmental contamination by TRWPs, the presence and extent of chemical contamination of milk by rubber-related chemicals remains unknown. This knowledge gap is particularly critical given the widespread global consumption of milk and the frequent proximity of dairy farms to busy roads, as well as the widespread use of rubber pipes and other parts in the dairy industry. In this study, we examined the presence of rubber-related compounds in milk by analyzing 17 samples collected from Swiss dairy farms located in different settings, including alpine farms and farms located near roads with varying traffic densities. While previous studies have examined chemical migration from food contact materials in general, we did not identify any work specifically addressing rubber-derived contaminants in dairy production environments. To further elucidate potential contamination pathways, we additionally analyzed rubber components used during milk production and handling to assess whether these materials could constitute a direct source of rubber-related compounds in milk. By combining environmental exposure assessment with an evaluation of production-related materials, this study provides new insights into possible sources of rubber-associated contaminants in dairy products. Overall, our findings contribute to a more comprehensive understanding of the environmental and public health implications of rubber-related pollutants within the food supply chain.

## 2 Materials & methods

### 2.1 Sampling and sample preparation

**Fig. 1:**
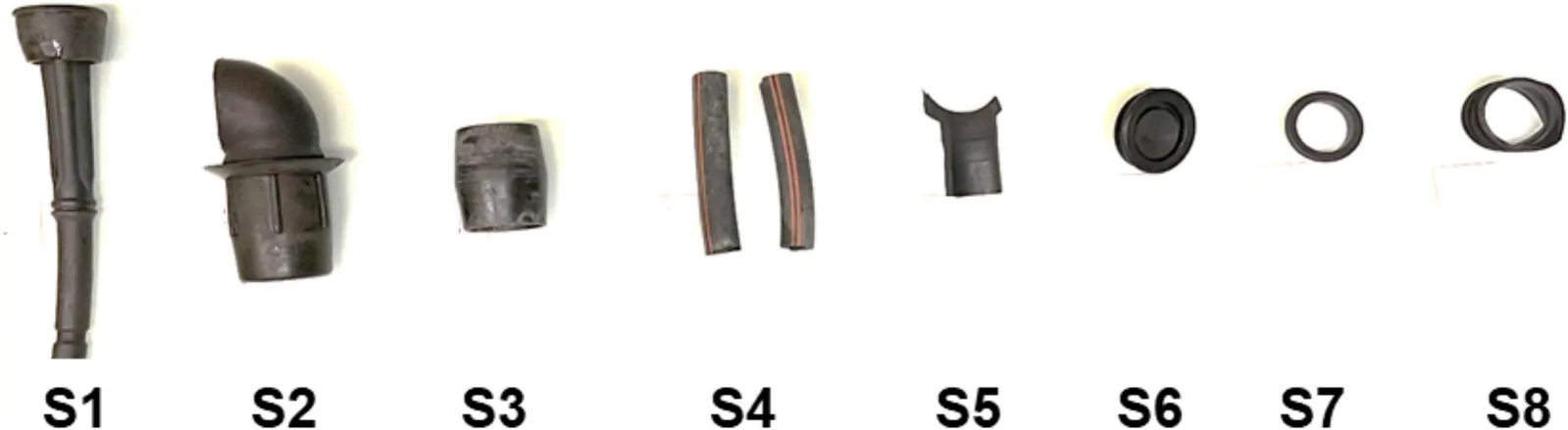
Rubber components used in milk production and analyzed in this study. (S1) teatcup liner, (S2) elbow connector, (S3) shirt rubber sleeve, (S4) rubber tubing, (S5) rubber connector, (S6) rubber plug, (S7) rubber tubing and (S8) larger rubber tubing. Components S1–S8 represent rubber parts that are in direct contact with milk during milking, transfer or temporary storage.

Seventeen raw cow milk samples were collected from 16 farms in Switzerland directly in the milk tank connected to the milking machine. This includes alpine farms (*n* = 3, samples M1-M3) and herd of cows grazing near a highway (*n* = 3, ∼50,000–150,000 cars/days (Federal Statistical Office), samples M10-M12), departmental road (*n* = 4, ∼3,000–30,000 cars/days (Federal Statistical Office), samples M4, M5, M13 and M14) and country roads (*n* = 6, ∼500–5000 cars/days (Federal Statistical Office), samples M6-M9, M15 and M16). One sample is a mix of raw milk from 4 farms (M17). This mixture of milk contains 57% of milk from farms located near country roads with a low traffic density and 43% from farms next to a departmental road with a medium to high traffic density. The samples were stored in falcon tubes of 50 mL at 4 °C until analysis. 3 g of milk were transferred in 50 mL tubes and doped with 50 μL of a mix internal standards before extraction (i.e. aniline-d5, benzothiazole-d4, DPG-d10, 6-PPDQ-d5, and DCU-d10). 1 g of NaCl were added to the samples before extraction with 6 mL of acetonitrile. The samples were stirred using an orbital shaker during 10 min and then centrifugated at 3000 rpm (relative centrifugal force: 1811) during 10 min. The extraction was repeated twice. Finally, the samples were evaporated under nitrogen flow to reduce the volume to 0.5 mL. The target compounds are low-volatility species (vapor pressure: ∼10^−6^–10^−3^ Pa); therefore, the loss due to evaporation is considered minimal. Different rubber components (*n* = 8) from milking equipment and other dairy production systems were examined to evaluate their potential contribution to milk contamination (Fig. 1). For determination of the total rubber-additive concentration, approximately 0.2 g of material was scraped from the components, spiked with an internal standard, and subjected to Soxhlet extraction during 16 h with methanol (MeOH) followed by a second extraction with dichloromethane (DCM) during 16 h. (Masset et al., 2022) The combined extract was concentrated to 0.5 mL, diluted as required, and analyzed by liquid chromatography tandem mass spectrometry (LC–MS/MS). To estimate surface-available concentrations, the interior surfaces of the components were rinsed by repeated (10 cycles) contact with 20 mL MeOH using a Pasteur pipette, the extract was spiked with internal standard and concentrated to 0.5 mL. After extraction all samples were analyzed by LC-MS/MS for the quantification the rubber-related compounds listed in Table S1 (Supporting information). The present study was designed to provide an initial indication of the occurrence and surface availability of rubber-related compounds in the investigated materials rather than to perform a formal migration assessment. The applied extraction and rinsing procedures represent exhaustive or semi-exhaustive approaches and do not reflect realistic conditions of use for milk-contact materials.

### 2.2 Analytical method

LC-MS/MS analyses were carried out with an ACQUITY UPLC system that was coupled to a Xevo TQ MS mass spectrometer equipped with an electrospray ionization source (ESI) (Waters Corp., Milford, USA). Chromatographic separation was performed with an ACQUITY UPLC HSS T3 column (100 × 2.1 mm, 1.8 μm) at a flow rate of 0.40 mL/min and a column temperature of 30 °C. The mobile phase consisted of (A) 0.1% v/v formic acid in 95% v/v water and 5% v/v acetonitrile and (B) 0.1% v/v formic acid in 95% v/v acetonitrile and 5% v/v water. The elution gradient used is presented in Table S2 in supporting information. The mass spectrometer parameters were set as follows; electrospray in positive mode, source temperature: 150 °C, desolvation temperature 600 °C, cone gas flow (N_2_): 50 L/h, desolvation gas flow: 1000 L/h, collision gas flow: 0.15 mL/min, ion spray voltage: 1500 V, cone voltage: 30 V. The optimal instrumental parameters for each analyte were obtained by tuning using direct infusion. Quantitative analyses were performed in MS/MS mode. Identification of the analytes was based on comparison of one or two MRM transitions and retention times. The analyte specific parameters are presented in Table S3 in supporting information. Data was acquired and processed with MassLynx V4.2 (Waters Corp., Milford, USA).

### 2.3 Quality assurance and control

Deuterated analytical standards (aniline-d5, benzothiazole-d4, DPG-d10, 6-PPDQ-d5, and DCU-d10) were selected as internal standards for each analyte based on their structural similarity and physicochemical properties (Table S3, supporting information). Internal standards were added to all samples prior to extraction to correct for potential matrix effects, analyte losses during sample preparation, and instrumental variability. All reported concentrations were normalized to the corresponding internal standard and subsequently corrected using analyte-specific recovery rates. Procedural blanks were included in all analytical sequences, and concentrations measured in blanks were subtracted from sample concentrations to account for background contamination. Calibration curves were constructed using matrix-matched standards covering the linear range of each analyte, and calibration standards were analyzed at the beginning and end of each analytical sequence to verify instrument stability and method performance. The limit of quantification (LOQ) was defined as the lowest calibration standard concentration within the linear range that met the predefined criteria for accuracy and precision. Method recovery was evaluated by spiking milk samples with known amounts of the target analytes prior to extraction. Spiked concentrations ranged from 1.7 to 5.5 ng/mL for all analytes except benzothiazole (BTH), which was spiked at 22.7 ng/mL. Recovery rates ranged from 47.1% to 159.0%, reflecting differences in extraction efficiency and matrix interactions among analytes. Because milk is a complex matrix, analytes exhibited compound-specific ion suppression or enhancement. Matrix-matched calibration together with isotope-labelled internal standards minimized these effects. Nevertheless, recoveries outside the ideal range indicate that reported concentrations should be regarded as semi-quantitative, particularly for analytes exhibiting the highest recoveries. These recovery rates were used to correct measured concentrations in all samples. Quality control samples, including spiked samples and blanks, were analyzed throughout each batch to ensure data reliability and consistency.

## 3 Results and discussion

### 3.1 Rubber-related compounds in milk samples

**Fig. 2:**
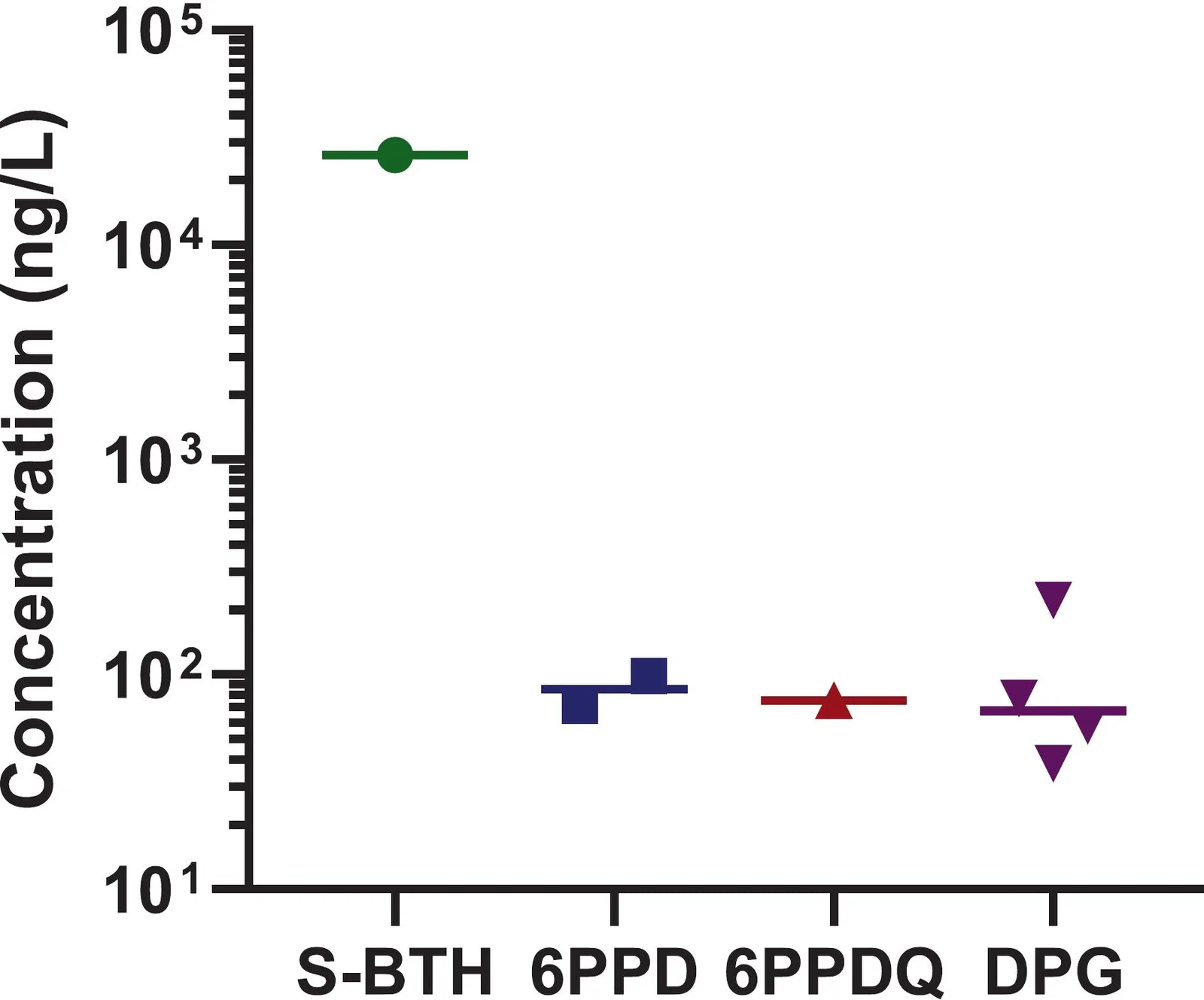
Concentrations of rubber-related chemicals in seven whole milk samples (*n* = 17 samples of which 7 showed detectable concentrations).

Seventeen milk samples were analyzed according to the protocol described previously. Among these samples, only four compounds, i.e. S-BTH, DPG, 6-PPD and 6-PPD-Q, were detected and quantified (Fig. 2**,** supporting information Table S4 and Table S5). In 41% of the samples analyzed in this study, at least one of these compounds was present at a concentration higher than the LOQ. S-BTH was detected in only one sample at a concentration of 26,258.5 ng/L. The elevated S-BTH concentration represents a clear outlier. Possible explanations include localized environmental contamination or release from a specific rubber component. Additional sampling would be required to distinguish among these hypotheses. DPG was detected in 23% of the milk samples with a concentration ranging from 38.8 to 223.3 ng/L (median: 68.0 ng/L). 6-PPD was measured in two milk samples (12% of the samples) at concentration ranging from 71.4 to 99.1 ng/L (median: 85.3 ng/L). Finally, 6-PPDQ was detected in only one sample at a concentration of 75.6 ng/L. No rubber-related chemicals were detected in milk samples from the three alpine farms located away from major road traffic (Table S5). In contrast, DPG, 6-PPD and/or 6-PPD-Q were detected in samples collected from farms located near country, departmental, and highway roads. However, no clear relationship was observed between either the detection frequency or measured concentrations of these compounds and road traffic intensity. In particular, several positive samples originated from farms located near country roads, which represented the lowest traffic category considered in this study. Given the limited number of samples within each road category, these results do not allow an association between traffic intensity and milk contamination to be established. Although the absence of detectable compounds in samples from alpine farms is compatible with lower environmental exposure to road-related emissions (Zhang et al., 2018), alpine and non-alpine farms may also differ in other factors, including feeding practices, husbandry conditions, and milking equipment. These factors were not systematically characterized and therefore represent potential confounding variables. Additional studies combining a larger number of farms with measurements of rubber-related chemicals in environmental matrices such as air, soil, and feed, as well as detailed information on farming practices and milking equipment, would be required to determine the contribution of road traffic to milk contamination. In addition to environmental sources, rubber components from milking equipment and other dairy production systems may represent another potential source of these compounds through migration into milk. In the following section, we explore this hypothesis by analyzing chemical additives in rubber components used in milk production.

### 3.2 Chemical additives in rubber components used in milk production

**Fig. 3:**
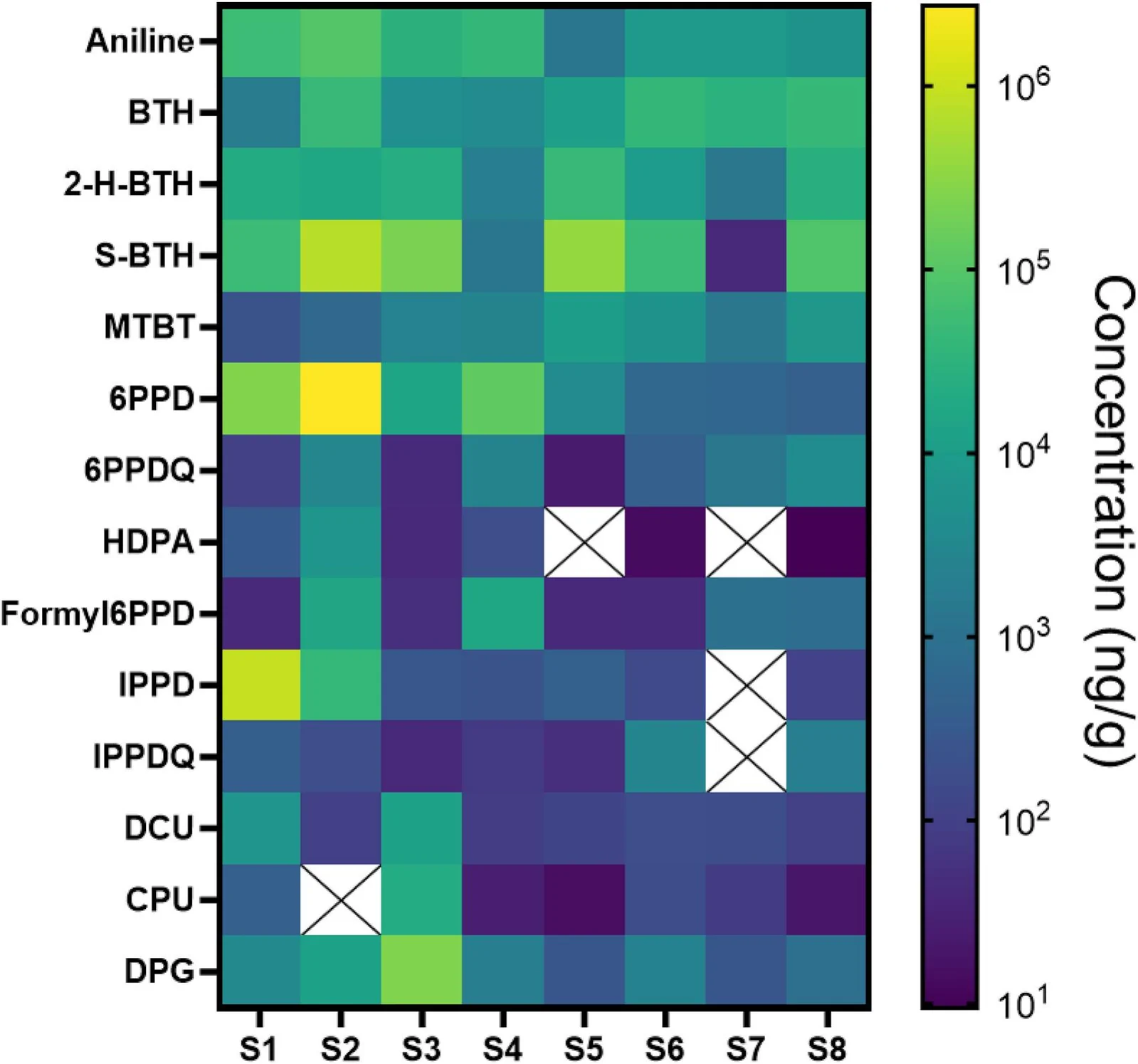
Heat-map of the bulk concentrations of rubber-related chemicals in the eight rubber components (S1 to S8) used in milk production. Compounds below the limit of quantification are represented by a white square with a cross.

**Fig. 4:**
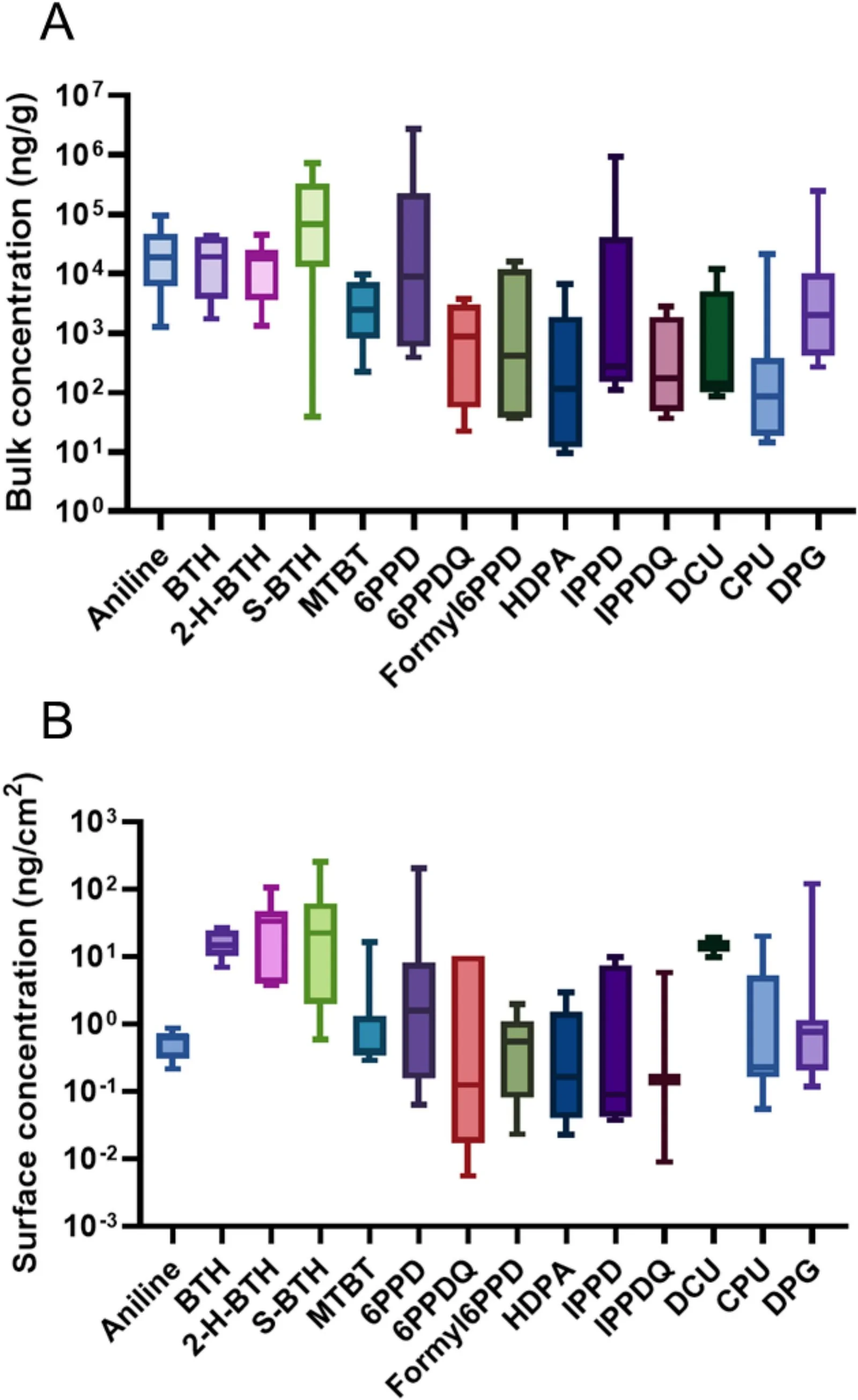
(A) Bulk and (B) surface concentration of rubber-related chemicals in the eight rubber components used in milk production.

Fourteen rubber-related chemicals were detected in almost all rubber components analyzed in this study and that are in direct contact with the milk (Fig. 3, supporting information Table S6). These analyses revealed that these materials can contain high levels of rubber-related chemicals similar to those used in car tires.(Wang et al., 2025) The total bulk concentration of these different compounds range between 42.9 μg/g in sample S7 up to 3.7 mg/g in sample S8 with an average total concentration of 817 μg/g (*n* = 8). One of most abundant chemicals detected in those samples is S-BTH (from 49 μg/g to 715 μg/g) which is typically used as rubber vulcanization accelerator to improve the efficiency, elasticity, tensile strength, and aging resistance of natural and synthetic rubber products (Fig. 4a) (R.T. Vanderbilt, Company, 1990). BTH and 2-H-BTH were also detected in all samples. Benzothiazoles are essential for controlling the sulfur vulcanization process. 2-H-BTH specifically arises as a by-product from the transformation of other benzothiazole-based additives used to prevent rubber aging (R.T. Vanderbilt, Company, 1990). They function, along with related compounds like MTBT, also detected in all rubber components, to ensure efficient sulfur vulcanization, improving the elasticity, strength, and durability of rubber products. High concentrations of 6-PPD (392 ng/g to 2.7 mg/g) and IPPD (109.9 ng/g to 921 μg/g) were also detected in the rubber components. These compounds are antioxidants and antiozonants widely used in rubber materials to prevent degradation, cracking, and loss of elasticity caused by heat, oxygen, and ozone exposure (R.T. Vanderbilt, Company, 1990). In contrast, their oxidation by-products, 6-PPDQ, formyl-6-PPD, HDPA, and IPPDQ, were detected at substantially lower concentrations. Concentrations of DPG ranging from 269 ng/g to 247 μg/g. DPG is primarily used as a secondary accelerator in the sulfur vulcanization of natural and synthetic rubbers to speed up curing times and enhance material properties. Aniline was detected at relatively high concentrations (from 1.3 μg/g to 96 μg/g) in all samples primarily because it is a key degradation by-product of guanidine-based vulcanization accelerators such as DPG and antioxidants such as 6-PPD and IPPD. Finally, CPU and DCU were also detected in almost all samples. These cyclic amines are in general part of the complex chemical mixture added to ensure tire longevity, durability, and resistance to degradation. The experiments on surface solubilization using MeOH demonstrated that certain components can be partially leached from rubber elements upon contact with an organic solvent (Fig. 4b). Although MeOH does not represent the fraction that would realistically be extracted by milk, this approach can be considered a worst-case scenario, providing an upper-bound estimate of potential compound release. The results therefore suggest a theoretical potential for migration through interaction with rubber components used in milking equipment and other dairy production systems. Moreover, the chemical profiles of the compounds leaching from the rubber elements were very similar to those measured in the bulk material, indicating that the substances released under these conditions largely reflect the composition of the rubber rather than selective extraction of surface-specific constituents.

### 3.3 Regulatory context

Materials based on natural and synthetic rubber are widely used in food contact applications, such as gaskets, tubing, seals, and gloves, due to their flexibility, resilience, and chemical resistance. However, these materials often contain a variety of additives, including vulcanization accelerators, antioxidants, plasticizers, and processing aids, that may migrate into food under certain conditions of use (e.g., temperature, duration of contact, fat content). Such migration may represent a potential source of chemical contamination and therefore requires careful assessment within the applicable regulatory framework. According to the recommendations on food contact materials XXI/1 of the German Federal Institute for Risk Assessment (Bundesinstitut für Risikobewertung; BfR), commodities based on natural and synthetic rubber intended for contact with food are classified into four categories depending on the duration and intensity of contact: long-term contact (category 1), medium-term contact (category 2), short-term contact (category 3), and contact of insignificant duration (category 4). Rubber components used in milking equipment, such as teat liners in milking systems, milking machine hoses, and seals for milk processing machines, are generally assigned to category 3, corresponding to short-term contact with food. In the present study, fourteen compounds were quantified in rubber components used in milk production. These substances included selected vulcanization accelerators, degradation products, and antioxidants commonly employed in rubber formulations. A French decree dated 5 August 2020 relating to rubber components intended to come into contact with foodstuffs requires the determination of specific migration limits for DPG and 6-PPD (Note, n.d.). Furthermore, in its recommendations for category 3 rubber materials, the BfR specifies that the content of 6-PPD, DPG and S-BTH in rubber components should not exceed 1.5%, 0.3% and 1%, respectively. (https://empfehlungen.bfr.bund.de/recommendations/211/notifications, 2026) After contact with milk or water at 40 °C for 10 min, the content of 6-PPD in these liquids should not exceed 0.3 mg/L. Maximum transfer of S-BTH into food should not exceed 2 mg/kg. Since no migration test was conducted in this study, compliance with migration limits cannot be claimed. Moreover, to our knowledge, no specific compositional limits or migration criteria are currently provided for the other eleven compounds detected in the investigated rubber components. Notably, 6-PPDQ, a transformation product of 6-PPD, was directly detected in certain milk samples. The occurrence of this compound in milk highlights the need for further toxicological evaluation and regulatory consideration, particularly in view of its potential formation during processing, aging, or oxidative degradation of rubber materials under real-use conditions.

## 4 Conclusion and perspectives

Although this study does not account for potential daily and seasonal variability specific to each farm, it demonstrates that selected rubber-related chemicals, including benzothiazoles, guanidine-based accelerators, and *p*-phenylenediamine antioxidants, can be detected in raw milk at trace levels and are present in rubber components used throughout dairy production systems. The absence of detectable target compounds in samples from the three alpine farms, compared with their occurrence in several non-alpine farms, suggests that differences in environmental exposure and/or farming practices may influence milk contamination. However, no clear relationship between detection frequency or concentration and road traffic intensity was observed, and the limited sample size and potential differences in farm characteristics preclude attribution of the observed contamination to road traffic. The high concentrations of several of the same additives in rubber materials in direct contact with milk indicate that dairy equipment represents another plausible contamination source. Surface solubilization experiments further demonstrate the availability of these compounds for release from rubber materials, although the experimental conditions used here do not represent realistic milk-contact conditions. Although the measured concentrations in milk were generally low, the presence of multiple rubber-related additives highlights the need for a better understanding of their migration mechanisms, cumulative exposure, and potential toxicological relevance. These findings highlight the need for further toxicological assessment and regulatory consideration of rubber-contact materials used in food production systems to better evaluate potential chronic dietary exposure and associated public health risks. Future work should integrate daily and seasonal environmental monitoring (e.g. air, soil, and feed), migration studies under realistic milking conditions, and targeted risk assessments to disentangle environmental versus equipment-related sources and to support the development of safer materials for food-contact applications in the dairy sector.

## CRediT authorship contribution statement

**Florian Breider:** Writing – review & editing, Writing – original draft, Visualization, Validation, Supervision, Resources, Project administration, Methodology, Investigation, Funding acquisition, Data curation, Conceptualization. **Dominique Grandjean:** Writing – review & editing, Supervision, Methodology, Investigation, Formal analysis. **Claire Andrey:** Writing – review & editing, Methodology, Investigation, Formal analysis. **Thibault Masset:** Writing – review & editing, Methodology, Investigation. **Beat J. Brüschweiler:** Writing – review & editing, Writing – original draft, Investigation, Conceptualization.

## Declaration of competing interest

The authors declare the following financial interests/personal relationships which may be considered as potential competing interests: Florian Breider reports financial support was provided by Federal Food Safety and Veterinary Office (FSVO), Switzerland. If there are other authors, they declare that they have no known competing financial interests or personal relationships that could have appeared to influence the work reported in this paper.

## Data Availability

Data will be made available on request.

## Appendix A Supplementary data

Supplementary material Additional analytical parameters and raw data a provided in Supplementary data
