# Supplementary material for: Rubber-related chemical contamination in milk: Implications for dairy production systems
Source: Food Chem X. 2026 Aug 29;39:104373. doi: 10.1016/j.fochx.2026.104373 (PMC13562402; doi:10.1016/j.fochx.2026.104373)
Supplement: Supplementary file 1 [file mmc1.docx]

**Supporting Information**

**Food Chemistry X**

**Rubber-Related Chemical Contamination in Milk: Implications for Dairy Production Systems**

Florian Breider ^a*^, Dominique Grandjean ^a^, Claire Andrey ^a^, Thibault Masset ^a^‬‬, Beat J. Brüschweiler ^b^

^a^ Ecole Polytechnique Fédérale de Lausanne (EPFL), Central Environmental Laboratory, Station 2, CH-1015 Lausanne, Switzerland

^b^ Federal Food Safety and Veterinary Office (FSVO), Knowledge Foundations Division, Bern, Switzerland

*Corresponding author: Florian Breider; email: florian.breider@epfl.ch

**Table S1.** List of compounds analyzed by LC-MS/MS

| Abbreviation | IUPAC name | CAS n° |
| --- | --- | --- |
| AN | Aniline | 62-53-3 |
| 6PPD | *N*-(1,3-dimethylbutyl)-N′-phenyl-1,4-phenylenediamine | 793-24-8 |
| 6PPD-Q | 2-((4-methylpentan-2-yl)amino)-5-(phenylamino)cyclohexa-2,5-diene-1,4-dione | 2754428-18-5 |
| HDPA | 4-Hydroxydiphenylamine | 122-37-2 |
| DPG | 1,3-diphenylguanidine | 102-06-7 |
| HMMM | hexa(methoxymethyl)melamine | 3089-11-0 |
| BTH | benzothiazole | 95-16-9 |
| MTBT  S-BTH | 2-methylthiobenzothiazole  2-mercaptobenzothiazole | 615-22-5  149-30-4 |
| 2-H-BTH | 2-hydroxybenzothiazole | 934-34-9 |
| TMQ | 1,2-dihydro-2,2,4-trimethylquinoline | 26780-96-1 |
| CPU | 1-cyclohexyl-3-phenylurea | 886-59-9 |
| DCU | 1,3-dicyclohexylurea | 2387-23-7 |
| IPPD | N-isopropyl-N'-phenyl-p-phenylenediamine | 101-72-4 |
| Formyl-6PPD | N-Formyl-6PPD | / |
| IPPD-Q | 2-[(1-methylethyl)amino]-5-(phenylamino)-2,5-cyclohexadiene-1,4-dione | 68054-73-9 |

**Table S2.** Elution gradient of the liquid chromatographic column with solvents A: 0.1 % formic acid in 95% water and 5% acetonitrile and B: 0.1% formic acid in 95% acetonitrile and 5% water

| Step | Time (min) | Flow (mL/min) | %A | %B |
| --- | --- | --- | --- | --- |
| 1 2 3 4 5 6 | 0.00  0.50 5.00 9.00 10.00 12.00 | 0.40  0.40  0.40  0.40  0.40  0.40 | 95.0  95.0  5.0  5.0  95.0  95.0 | 5.0  5.0  95.0  95.0  5.0  5.0 |

**Table S3.** Compounds specific ionization and acquisition parameters measured in this study and used for quantification with UPLC-MS/MS. The LOQs are expressed in ng of analyte per gram. BTH-d4 was used as an internal standard for BTH, 2-H-BTH, S-BTH, MTBT; AN-d5 was used as an internal standard for AN, DPG-d10 was used as an internal standard for DPG, DCU-d10 was used as an internal standard for DCU and CPU; 6-PPD-Q-D5 was used as an internal standard for 6-PPD, 6-PPD-Q, HDPA, IPPD, IPPD-Q, Formyl-6-PPD, TMQ, HMMM.

**Table S4**. Concentrations of rubber-related chemicals in whole milk samples (*n*=17 samples). Sample M17 is a mixture of raw milk collected from 4 farms. Therefore, this sample cannot be compared directly to single-farm samples.

| ng/L milk | M1 | M2 | M3 | M4 | M5 | M6 | M7 | M8 | M9 | M10 | M11 | M12 | M13 | M14 | M15 | M16 | M17 |
| --- | --- | --- | --- | --- | --- | --- | --- | --- | --- | --- | --- | --- | --- | --- | --- | --- | --- |
| AN | <LOQ | <LOQ | <LOQ | <LOQ | <LOQ | <LOQ | <LOQ | <LOQ | <LOQ | <LOQ | <LOQ | <LOQ | <LOQ | <LOQ | <LOQ | <LOQ | <LOQ |
| BTH | <LOQ | <LOQ | <LOQ | <LOQ | <LOQ | <LOQ | <LOQ | <LOQ | <LOQ | <LOQ | <LOQ | <LOQ | <LOQ | <LOQ | <LOQ | <LOQ | <LOQ |
| 2-H-BTH | <LOQ | <LOQ | <LOQ | <LOQ | <LOQ | <LOQ | <LOQ | <LOQ | <LOQ | <LOQ | <LOQ | <LOQ | <LOQ | <LOQ | <LOQ | <LOQ | <LOQ |
| S-BTH | <LOQ | <LOQ | <LOQ | 26’258.5 | <LOQ | <LOQ | <LOQ | <LOQ | <LOQ | <LOQ | <LOQ | <LOQ | <LOQ | <LOQ | <LOQ | <LOQ | <LOQ |
| MTBT | <LOQ | <LOQ | <LOQ | <LOQ | <LOQ | <LOQ | <LOQ | <LOQ | <LOQ | <LOQ | <LOQ | <LOQ | <LOQ | <LOQ | <LOQ | <LOQ | <LOQ |
| DPG | <LOQ | <LOQ | <LOQ | <LOQ | 38.8 | 77.7 | 58.3 | <LOQ | <LOQ | <LOQ | 223.3 | <LOQ | <LOQ | <LOQ | <LOQ | <LOQ | <LOQ |
| 6-PPD | <LOQ | <LOQ | <LOQ | <LOQ | <LOQ | <LOQ | <LOQ | <LOQ | <LOQ | <LOQ | <LOQ | <LOQ | <LOQ | <LOQ | 71.4 | 99.1 | <LOQ |
| 6-PPDQ | <LOQ | <LOQ | <LOQ | <LOQ | <LOQ | 75.6 | <LOQ | <LOQ | <LOQ | <LOQ | <LOQ | <LOQ | <LOQ | <LOQ | <LOQ | <LOQ | <LOQ |
| TMQ | <LOQ | <LOQ | <LOQ | <LOQ | <LOQ | <LOQ | <LOQ | <LOQ | <LOQ | <LOQ | <LOQ | <LOQ | <LOQ | <LOQ | <LOQ | <LOQ | <LOQ |
| HMMM | <LOQ | <LOQ | <LOQ | <LOQ | <LOQ | <LOQ | <LOQ | <LOQ | <LOQ | <LOQ | <LOQ | <LOQ | <LOQ | <LOQ | <LOQ | <LOQ | <LOQ |
| Formyl-6PPD | <LOQ | <LOQ | <LOQ | <LOQ | <LOQ | <LOQ | <LOQ | <LOQ | <LOQ | <LOQ | <LOQ | <LOQ | <LOQ | <LOQ | <LOQ | <LOQ | <LOQ |
| CPU | <LOQ | <LOQ | <LOQ | <LOQ | <LOQ | <LOQ | <LOQ | <LOQ | <LOQ | <LOQ | <LOQ | <LOQ | <LOQ | <LOQ | <LOQ | <LOQ | <LOQ |
| DCU | <LOQ | <LOQ | <LOQ | <LOQ | <LOQ | <LOQ | <LOQ | <LOQ | <LOQ | <LOQ | <LOQ | <LOQ | <LOQ | <LOQ | <LOQ | <LOQ | <LOQ |

**Table S5**. Relationship between each individual sample in which target compounds were detected and the traffic conditions.

| Sample | Detected compounds | Traffic conditions |
| --- | --- | --- |
| M4 | S-BTH | departmental road (~3’000-30’000 cars/days) |
| M5 | DPG | departmental road (~3’000-30’000 cars/days) |
| M6 | DPG, 6-PPDQ | country road (~500-5’000 cars/days) |
| M7 | DPG | country road (~500-5’000 cars/days) |
| M11 | DPG | highway (~50’000-150’000 cars/days) |
| M15 | 6-PPD | country road (~500-5’000 cars/days) |
| M16 | 6-PPD | country road (~500-5’000 cars/days) |

|  | S1 | | S2 | | S3 | | | S4 | | | | S5 | | | | S6 | | | | S7 | | | | S8 | | | |  |
| --- | --- | --- | --- | --- | --- | --- | --- | --- | --- | --- | --- | --- | --- | --- | --- | --- | --- | --- | --- | --- | --- | --- | --- | --- | --- | --- | --- | --- |
|  | [ng/g] | [ng/cm²] | [ng/g] | [ng/cm²] | [ng/g] | [ng/cm²] | | [ng/g] | | [ng/cm²] | | [ng/g] | | [ng/cm²] | | [ng/g] | | [ng/cm²] | | [ng/g] | | [ng/cm²] | | [ng/g] | | [ng/cm²] | |  |
| AN | 5.1E+04 | 7.0E-01 | 9.6E+04 | 6.6E-01 | 2.9E+04 | 6.6E-01 | | 3.9E+04 | | 6.6E-01 | | 1.3E+03 | | 2.2E-01 | | 8.5E+03 | | 3.4E-01 | | 8.3E+03 | | 8.6E-01 | | 5.6E+03 | | 3.0E-01 | |  |
| 2-H-BTH | 2.1E+04 | 3.8E+00 | 1.5E+04 | 3.3E+01 | 2.2E+04 | 4.5E+01 | | 1.9E+03 | | 3.3E+01 | | 4.5E+04 | | 1.1E+02 | | 8.7E+03 | | 4.9E+01 | | 1.3E+03 | | 3.8E+00 | | 2.6E+04 | | 4.6E+00 | |  |
| S-BTH | 4.9E+04 | 4.3E+01 | 7.1E+05 | 5.0E+00 | 2.2E+05 | 4.0E+01 | | 1.3E+03 | | 5.0E+00 | | 3.7E+05 | | 2.5E+02 | | 5.0E+04 | | 6.8E+01 | | 3.9E+01 | | 9.9E-01 | | 8.4E+04 | | 5.9E-01 | |  |
| MTBT | 2.2E+02 | 3.4E-01 | 6.2E+02 | 3.9E-01 | 2.4E+03 | 2.9E-01 | | 2.6E+03 | | 3.9E-01 | | 9.8E+03 | | 1.6E+01 | | 5.6E+03 | | 1.3E+00 | | 1.4E+03 | | nd | | 7.6E+03 | | 3.8E-01 | |  |
| 6PPD-Q | 1.0E+02 | 1.2E+00 | 3.1E+03 | 1.0E+01 | 4.0E+01 | 1.7E-02 | | 2.6E+03 | | 1.0E+01 | | 2.2E+01 | | nd | | 4.0E+02 | | 1.2E-01 | | 1.4E+03 | | 9.2E-02 | | 3.8E+03 | | 5.6E-03 | |  |
| DCU | 6.7E+03 | nd | 1.0E+02 | 1.0E+01 | 1.2E+04 | 1.9E+01 | | 8.7E+01 | | nd | | 1.2E+02 | | nd | | 1.8E+02 | | nd | | 1.7E+02 | | nd | | 1.0E+02 | | nd | |  |
| CPU | 3.9E+02 | nd | <loq | 2.5E-01 | 2.1E+04 | 2.0E+01 | | 2.7E+01 | | nd | | 1.4E+01 | | 5.5E-02 | | 1.7E+02 | | 2.1E-01 | | 8.5E+01 | | 2.4E-01 | | 1.9E+01 | | 2.0E-01 | |  |
| 6PPD | 2.5E+05 | 2.0E+02 | 2.7E+06 | 1.6E-01 | 1.4E+04 | 6.4E+00 | | 1.3E+05 | | 1.6E-01 | | 3.7E+03 | | 8.1E-01 | | 6.1E+02 | | 8.8E+00 | | 5.8E+02 | | 2.3E+00 | | 3.9E+02 | | 6.4E-02 | |  |
| DPG | 3.5E+03 | 4.7E-01 | 1.3E+04 | 1.2E-01 | 2.5E+05 | 1.2E+02 | | 1.8E+03 | | 1.2E-01 | | 2.7E+02 | | 7.4E-01 | | 2.2E+03 | | 1.2E+00 | | 2.7E+02 | | 9.1E-01 | | 8.8E+02 | | 7.9E-01 | |  |
| HMMM | <loq | 5.3E-02 | <loq | nd | <loq | nd | | <loq | | nd | | <loq | | nd | | <loq | | nd | | <loq | | nd | | <loq | | nd | |  |
| BTH | 1.7E+03 | 9.4E+00 | 4.3E+04 | 2.6E+01 | 4.5E+03 | | 1.2E+01 | | 3.5E+03 | | 2.6E+01 | | 1.0E+04 | | 7.0E+00 | | 3.9E+04 | | 1.6E+01 | | 2.8E+04 | | 1.3E+01 | | 4.2E+04 | | 2.0E+01 | |
| TMQ | <loq | 9.4E-02 | <loq | nd | <loq | nd | | <loq | | nd | | <loq | | nd | | <loq | | nd | | <loq | | nd | | <loq | | nd | |  |
| IPPD | 9.2E+05 | 5.2E-01 | 4.1E+04 | 9.8E+00 | 2.7E+02 | 4.0E-02 | | 2.2E+02 | | 9.8E+00 | | 4.4E+02 | | 3.7E-02 | | 1.5E+02 | | 4.7E-02 | | <loq | | 7.3E-02 | | 1.1E+02 | | 1.1E-01 | |  |
| IPPDQ | 3.8E+02 | nd | 1.8E+02 | 5.8E+00 | 3.7E+01 | 9.0E-03 | | 7.4E+01 | | nd | | 4.8E+01 | | nd | | 2.8E+03 | | nd | | <loq | | nd | | 1.9E+03 | | 1.5E-01 | |  |
| HDPA | 3.1E+02 | 3.0E+00 | 6.7E+03 | 1.5E+00 | 3.8E+01 | 3.9E-02 | | 1.9E+02 | | 1.5E+00 | | <loq | | 2.3E-02 | | 1.3E+01 | | 2.6E-01 | | <loq | | 4.4E-02 | | 9.5E+00 | | 6.7E-02 | |  |
| Formyl6PPD | 3.8E+01 | 2.0E+00 | 1.5E+04 | 7.8E-01 | 5.3E+01 | 2.3E-02 | | 1.6E+04 | | 7.8E-01 | | 3.7E+01 | | nd | | 4.0E+01 | | 1.0E-01 | | 1.0E+03 | | 3.1E-01 | | 7.7E+02 | | nd | |  |

**Table S6**. Bulk and surface concentration of rubber-related chemicals in the eight rubber components used in milk production. nd = not detected, <loq = detected but below the limit of quantification.
